# The potential application of fermented tea as a drink for regulating bone mass

**DOI:** 10.3389/fphar.2024.1353811

**Published:** 2024-07-04

**Authors:** Qiaolu Xu, Yikang Yu, Ke Chen

**Affiliations:** ^1^ Department of Geriatric Medicine, The Second Hospital of Jinhua, Jinhua, China; ^2^ School of Pharmaceutical Sciences, Zhejiang Chinese Medical University, Hangzhou, China; ^3^ Orthopedics and Traumatology Department, The Second Affiliated Hospital, Zhejiang Chinese Medical University, Hangzhou, China

**Keywords:** fermented tea, osteoporosis, bone mass regulation, tea polyphenols, tea pigment

## Abstract

Currently, there is evidence to suggest the benefits of drinking fermented tea for people with osteoporosis, and based on this, many studies have been conducted on the dosage, exact ingredients, mechanisms, and industrial applications of fermented tea for protecting against osteoporosis. A summary and analysis of studies on the regulation of bone mass by oolong tea, black tea, and their active ingredients (including 39 known catechin compounds) was conducted. It was found that the regulation of bone mass by fermented tea is backed by evidence from epidemiology, animal experiments, and cell experiments. The main active components of fermented tea are tea polyphenols, tea pigments, and trace amino acids. The specific mechanisms involved include regulating bone marrow mesenchymal stem cell osteogenesis, inhibiting osteoclast activity, promoting calcium and phosphorus absorption, reducing inflammation levels, regulating gut microbiota, regulating endocrine function, and inhibiting oxidative stress. In terms of its application, extraction, precipitation, biosynthesis and membrane separation method are mainly used to separate the active ingredients of anti osteoporosis from fermented tea. In conclusion, fermented tea has sufficient theoretical and practical support for regulating bone mass and preventing osteoporosis, and is suitable for development as a health supplement. At the same time, a large amount of epidemiological evidence is needed to prove the specific dosage of tea consumption.

## Introduction

**FIGURE 1:**
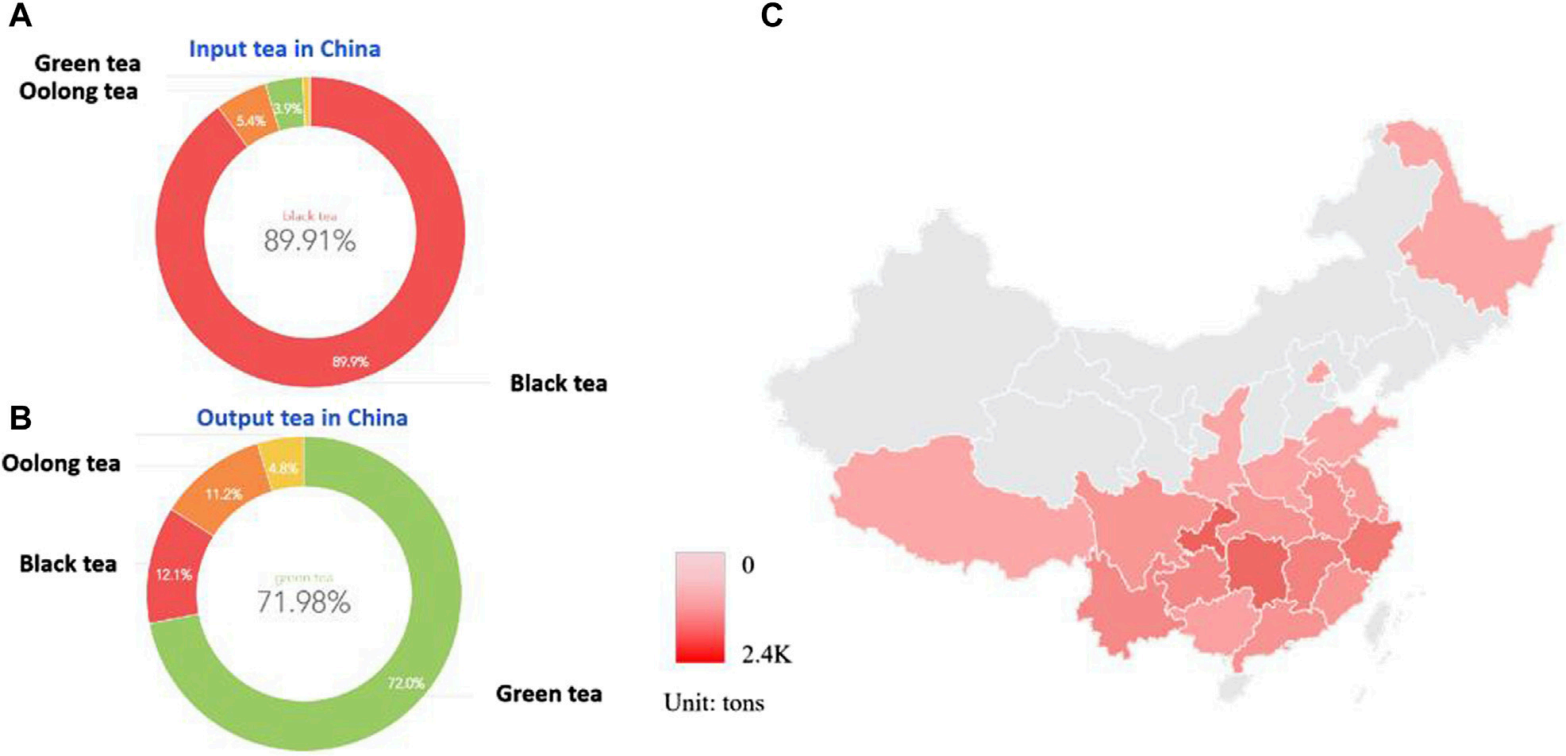
China as a major exporter and importer of tea **(A)**. Fermented tea accounts for the majority of China’s imported tea, with black tea accounting for 89.91% of the trade volume; **(B)**. Non-fermented tea accounts for the majority of China’s tea exports, with green tea accounting for 71.98% of the trade volume, black tea accounting for 11.79%, and oolong tea accounting for 11.19%. **(C)**. Schematic diagram of the main production areas and export volume of fermented tea in China, mainly concentrated in the Yangtze River Basin in the south.

Tea, as a multifunctional health drink, is one of the most widely consumed beverages in the world. It was consumed by more than two-thirds of the world’s population, second only to water in terms of popularity (Zhang et al., 2023). Taking China as an example, it is a major producer and consumer of tea. In traditional Chinese usage, tea is the bud and leaf of Camellia plants, and its production, cultivation, and distribution centers are mainly concentrated in the Yangtze River Basin. According to statistics from the China Association for the Promotion of International Agricultural Cooperation, China exported a total of 174,971 tons of tea from January to June 2023, with an export value of 800 million US dollars. Among them, green tea accounted for 71.98% of the trade value, black tea accounted for 11.79%, and oolong tea accounted for 11.19%; A total of 17,255 tons of tea were imported, with an import value of 65,643,390 US dollars, of which black tea accounted for 89.91% of the trade volume, and we can find the distribution of tea exports and imports by type in China through customs data (Figure 1). It can be seen that China is a major importer of fermented tea, and an exporter of non fermented tea. The active ingredients in tea include tea polyphenols, amino acids, tea polysaccharides, tea pigments, which have anti-aging, antiviral, anti-inflammatory, antioxidant, and anti osteoporosis effects, and have attracted increasing attention from consumers.

In the past 20 years, multiple epidemiological studies have shown that moderate consumption of tea products, especially fermented tea, can protect bone health. Subsequently, many studies have emerged on the effects of different tea extracts and a single component on bone metabolism. Fermented tea mainly includes oolong tea and black tea, which have been widely used in the production of functional drinks and health products in recent years (Yoo et al., 2023). When compared different types of tea, there have significant differences in compound composition and health functions, among which the research on the bone protective effects of fermented teas such as oolong tea and black tea has become a hot topic in recent years (Shen et al., 2023). This study systematically reviews the research results and application prospects of the active ingredients in fermented tea leaves of Camellia plants on bone mass regulation.

## Clinical and epidemiological evidence of the involvement of fermented tea in bone mass regulation

Drinking habit is an important aspect of health management in research on the prevention and treatment of osteoporosis. The impact of tea and coffee on bone density has been a hot topic in recent osteoporosis research years. Many epidemiological investigations have shown an association between daily tea (especially fermented tea) consumption and bone health. And the relationship between tea and bone health have attracted the attention of many national research institutions (Table 1). The earliest report by Wu in 2002 pointed out that elderly people in Taiwan who drink black tea or oolong tea once a week for at least 6 months can protect the bone mineral density (BMD) of the whole body, lumbar spine, femoral neck, and Ward’s triangle (Wu et al., 2002). Subsequent studies have confirmed this conclusion (Hirata et al., 2016; Liu et al., 2018; Li et al., 2020; Duan et al., 2020; Yu et al., 2020; Sun et al., 2021). In addition to maintaining bone density, a study conducted in Sweden in 2006 found that drinking black tea can reduce the risk of fractures (Hallstrom et al., 2006). Drinking fermented tea at the same time can maintain a relatively stable bone metabolism level in perimenopausal women, such as serum carboxy-terminal cross-linked telopeptide of type 1 collagen (CTX), procollagen type 1 N-terminal propeptide (PINP), and osteocalcin (OC) level (Sun et al., 2021). However, due to the limitations of epidemiology and clinical observation, as well as the influence of tea types and processing techniques in different regions, most of the above studies cannot provide specific dosage recommendations for daily consumption of fermented tea to protect bone mass. This has also led to *in vitro* and *in vivo* experimental studies on the effects of black tea and its active ingredients on bone metabolism.

**TABLE 1:** Summary of epidemiological studies on the use of fermented tea to protect bone mass.

References	Type of tea	Dosage and timing of use	Outcomes	Type of study	Country (region)
Wu et al., 2002	black and oolong tea	At least once a week for a minimum of 6 months	BMD for the whole body, lumbar spine, femur neck and Ward triangle	Prospective Studies	Taiwan, China
Hallstrom et al. (2006)	Black tea	<1 cup/day, 1 cup/day, 2–3 cups/day, ≥4 cups/day	Rate of fractures	Prospective Studies	Uppsala, Sweden
Hirata, H et al., 2016	black tea	120 mL 1cup/day, ≥2cups/day	lumbar spine and total hip BMD	cross-sectional study	Niigata, Japan
Liu et al., 2018	black tea	0∼1time/week, 2∼5times/week,> 5 times/week	L1∼4 and femoral neck bone density	cross-sectional study	Guangzhou, China
Li et al., 2018	black tea	Not elaborated	BMD	cross-sectional study	Yichun, China
Yu et al., 2020	black tea	≤3 times/week, >3 times/week	Neck, Ward triangle, trochanteric, shaft, total femur bone density	cross-sectional study	Beijing, China
Duan et al. (2020)	Oolong tea	1–5 cups/day, not >5 cups/day	calcaneus BMD	cross-sectional study	Shantou, China
Sun et al., 2021	black tea	0∼1time/week, 2∼5times/week,> 5 times/week	Female lumbar vertebrae 1–4 and femoral neck bone mineral density, serum CTX, PINP and OC	cross-sectional study	Huzhou, China

BMD, bone mineral density; PINP, procollagen type 1 N-terminal propeptide, CTX, carboxy-terminal cross-linked telopeptide of type 1 collagen; OC, osteocalcin.

## Experimental study on the direct involvment of fermented tea in bone mass regulation

Das was the first to use black tea water extract to intervene in a rat model of osteoporosis caused by bilateral ovariectomy. The study found that black tea aqueous extracts at a concentration of 2.5% and 1 mL/100 g for 28 days can significantly improve the bone density of the right femur, eighth thoracic rib, eighth thoracic vertebra, and fourth lumbar vertebra in ovariectomized rats (Das et al., 2004). Subsequently, Das confirmed that black tea extract at this dose concentration was beneficial for the elevation of serum estradiol while inhibiting osteoclast activity (Das et al., 2005). In addition, they also found that this extract can significantly promote intestinal calcium absorption (Das et al., 2013). Sun compared the effects of Pu’er tea, black tea, and green tea on the bone microstructure of ovariectomized osteoporosis rats. The results showed that black tea data was more stable, indicating that black tea has a more stable effect on improving bone microstructure than Pu’er tea and green tea (Sun, 2016). Shalan also showed similar results, with tests showing that the black tea aqueous extract is rich in catechins. It was also found that the extract had the best intervention effect on ovariectomized rats at a dose of 300 mg/kg, which is beneficial for bone regeneration and inhibits bone resorption (Shalan et al., 2017). Wang showed that ovariectomy (OVX) rats treated with black tea extract (BTE) for 12 weeks maintained their calcium (Ca) and phosphorus P) homeostasis and exhibited significantly enhanced levels of estradiol (E_2_) and Osteoprotegerin (OPG). At the same time, it also reduced the levels of interleukin-1β (IL-1β), interleukin-6 (IL-6) and improved the organ coefficient of uterus, BMD of the femur in OVX rats. In addition, this study confirmed that BTE inhibited receptor activator of nuclear factor kappa-B ligand (RANKL) stimulated osteoclast differentiation in RAW264.7 cells and effectively inhibited the expression of osteoclast related genes and proteins (Wang et al., 2018). These studies all suggest the bone conservation potential of black tea as a beverage and its advantages over non fermented tea. We summarize all the studies on the direct use of fermented tea extracts as follows (Table 2). However, only a few of these studies have delved into the specific compound composition that plays a role in regulating bone mass in fermented tea. Further research is needed to investigate the effects of active ingredient monomers and certain components in fermented tea.

**TABLE 2:** Summary of Experimental study on the direct involvement of fermented tea in bone mass regulation.

References	Extracts	Dosage and timing of use	Affected objects	Outcomes	Country (region)
Das et al., 2004	Black tea aqueous extract	2.5% aqueous BTE at a single dose of 1 mL/100 g body weight daily for 28 days	OVX rats	Improve the bone density	Calcutta, India
Das et al., 2005	Black tea aqueous extract	2.5% aqueous BTE at a single dose of 1 mL/100 g body weight daily for 28 days	OVX rats	Improve the serum estradiol while inhibiting osteoclast activity	Calcutta, India
Das et al., 2013	Black tea aqueous extract	2.5% aqueous BTE at a single dose of 1 mL/100 g body weight daily for 28 days	OVX rats	Promote intestinal calcium absorption	Calcutta, India
Sun, 2016	Pu-erh tea, black tea and green tea	0.1–0.9 g/kg/day for 12 weeks	OVX rats	Improve bone microstructure	Tangshan, China
Shalan et al., 2017	Black tea aqueous extract	250 mg/kg black-tea aqueous extract for 16 weeks	OVX rats	Inhibit bone resorption	Selangor, Malaysia
Wang et al., 2018	Black tea aqueous extract	0.4 g/kg body weight/day for 12 weeks	OVX rats	Reduce the levels of interleukin-1β, interleukin-6 and improved the organ coefficient of uterus, BMD of the femur	Kunming, China
Wang et al., 2018	A combination of DC powder and BTE	50 μg/mL and 100 μg/mL for 5 days	mouse macrophage cells	Inhibited the expression of osteoclast related genes and proteins	Kunming, China

BTE, black tea extract; DC, dendrobium candidum; OVX, ovariectomy; BMD, bone mineral density.

## Active ingredients related to bone mass regulation in fermented tea

### Polyphenolic substances participate in regulating bone mass

Tea polyphenols (TPs), also known as tea tannins, are a general term for polyphenolic compounds in tea, accounting for 15%–20% of the total weight of tea. Mainly including catechins, flavanols, flavonoids, etc. Compared to green tea, the content of tea polyphenols in fermented tea has decreased, but still remains at around 10%. There have been numerous reports on the direct use of unpurified polyphenols or catechins to intervene in osteoporosis. Shen reported that tea polyphenols, administered at a dosage of 400 mg/kg, exhibited a significant inhibitory effect on bone loss and effectively mitigated the degradation of bone microstructure in ovariectomized rats. They also proposed a possible way to prevent oxidative stress damage in bone reconstruction by increasing the activity of glutathione peroxidase (Shen et al., 2008). Meanwhile, they also demonstrated through human experiments that a daily intake of 500 mg of tea polyphenols combined with exercise is beneficial for maintaining bone mass in elderly women (Shen et al., 2009). Later on, they also demonstrated that this tea polyphenol had a similar bone protective effect on male rats with testicles removed (Shen et al., 2009). Tea polyphenols have also been reported by Shao to have a strong antioxidant ability to clear reactive oxygen species, thereby producing a protective effect on bone loss in ovariectomized, OVX rats (Shao et al., 2011). TPs can increase the antioxidant effects of superoxide dismutase-1 and adenosine triphosphate synthase, as well as reduce the estrogenic effects of catechol O-methyltransferase. Zhuang investigated the antioxidant effect of tea polyphenols on fracture healing in rats, and found that 1.0 g/kg tea polyphenols have a certain promoting effect in the early stage of fracture healing in rats, and its mechanism is closely related to the antioxidant properties of tea polyphenols (Zhuang et al., 2016). Shen also found that compared with the OVX group, intervention with TP (1% and 1.5%) reduced rat serum procollagen type 1 N-terminal propeptide at 3 and 6 months, C-terminal propeptide of type I collagen at 3 months, insulin-like growth factor-I at 6 months, and improved bone microstructure at different time points. Among them, 1.5% TPs had the best overall therapeutic effect at 3 months (Shen et al., 2019). Wang further studied the antioxidant effect of tea polyphenols on the expression of fibroblast growth factor 2 (FGF-2) in the fracture healing site of rats, and found that TPs at 200–400  mg/kg/d could promote fracture healing in rats. The mechanism may be related to increasing the expression of FGF-2 (Wang, 2020). Lao discovered that TPs possess a robust capacity for enhancing osteogenesis, whilst simultaneously suppressing the differentiation of human adipose-derived stem cells (hADSCs) into adipogenic lineages. This effect is achieved through the upregulation of the RUNX2-BMP2 mediated osteogenic pathway and the inhibition of PPAR-γ induced adipogenesis signaling (Lao et al., 2022). Sheng confirmed through a glucocorticoid induced Osteoporosis (OP) rat model that tea polyphenols may regulate bone metabolism indicators, alleviate oxidative stress, and inhibit osteoblast apoptosis by regulating the SIRT3/Bcl-2 signaling pathway, thereby providing a good protective effect on glucocorticoid induced OP (Sheng and Yin, 2022). Researchers confirmed that tea polyphenols activate Nrf2/Keap1/ARE pathway to play an anti oxidative stress role, and invented and prepared a hydrogel rich in tea polyphenols, which can alleviate intervertebral disc degeneration on the basis of improving drug use efficiency (Song, 2023). These studies elucidate the main role and mechanism of bone mass protection in fermented tea from the perspective of total tea polyphenol extracts (Table 3), but further research on individual components is needed.

**TABLE 3:** Summary of research on the anti osteoporosis effect of tea polyphenols.

References	Extracts	Dosage and timing of use	Affected objects	Outcomes	Country (region)
Shen et al., 2008	TPs	400 mg/kg for 16 weeks	OVX rats	Inhibit bone loss and alleviate the degradation of bone microstructure	Texas, United States
Shen et al., 2009	TPs	500 mg/day for 24 mouths	Postmenopausal women	Be beneficial for maintaining bone mass in elderly women	Texas, United States
Shen et al., 2011	TPs	0.5% TPs for 16 weeks	Rats with testicular excision	Bone protective effect on male rats	Texas, United States
Shao et al., 2011	TPs	0.5% TPs for 16 weeks	OVX rats	Antioxidant ability to clear reactive oxygen species, thereby producing a protective effect on bone loss	Texas, United States.
Zhuang et al., 2016	TPs	1.0 g/kg fo 7、14 days	OVX rats	Have a certain promoting effect in the early stage of fracture healing	Shenzhen, China
Shen et al., 2019	TPs	0.15%, 0.5%, 1%, and 1.5% (g/dL) TPs for 3 or 6 months	OVX rats	Reduced rat serum PINP, CTX, IGF-1, and improved bone microstructure	Texas, United States.
Wang, 2020	TPs	200–400 mg/kg/d for 2、4、6 weeks	OVX rats	Increasing the expression of FGF-2	Hunan, China
Lao et al., 2022	TPs	1 and 10 μg/mL for 21 days and 14 days	hADSCs	Promote osteogenesis, while inhibiting hADSC differentiation	Ultimo, Australia
Shen et al., 2022	TPs	25、100 mg/kg for 12 weeks	Glucocorticoid-induced OP rats	Regulate bone metabolism indicators, alleviate oxidative stress, and inhibit osteoblast apoptosis	Nanjing, China
Song, 2023	TPs	50uM-100uM for 1/2/4 weeks	Rats with degenerative discs	play an anti-oxidative stress role	Suzhou, China

TPs, Tea polyphenols; OVX, ovariectomy; PINP, procollagen type 1 N-terminal propeptide, CTX, carboxy-terminal cross-linked telopeptide of type 1 collagen; IGF-1, insulin-like growth factor-I; FGF-2, fibroblast growth factor-2; hADSC, human adipose-derived stem cell.

## Catechins participate in regulating bone mass

**FIGURE 2:**
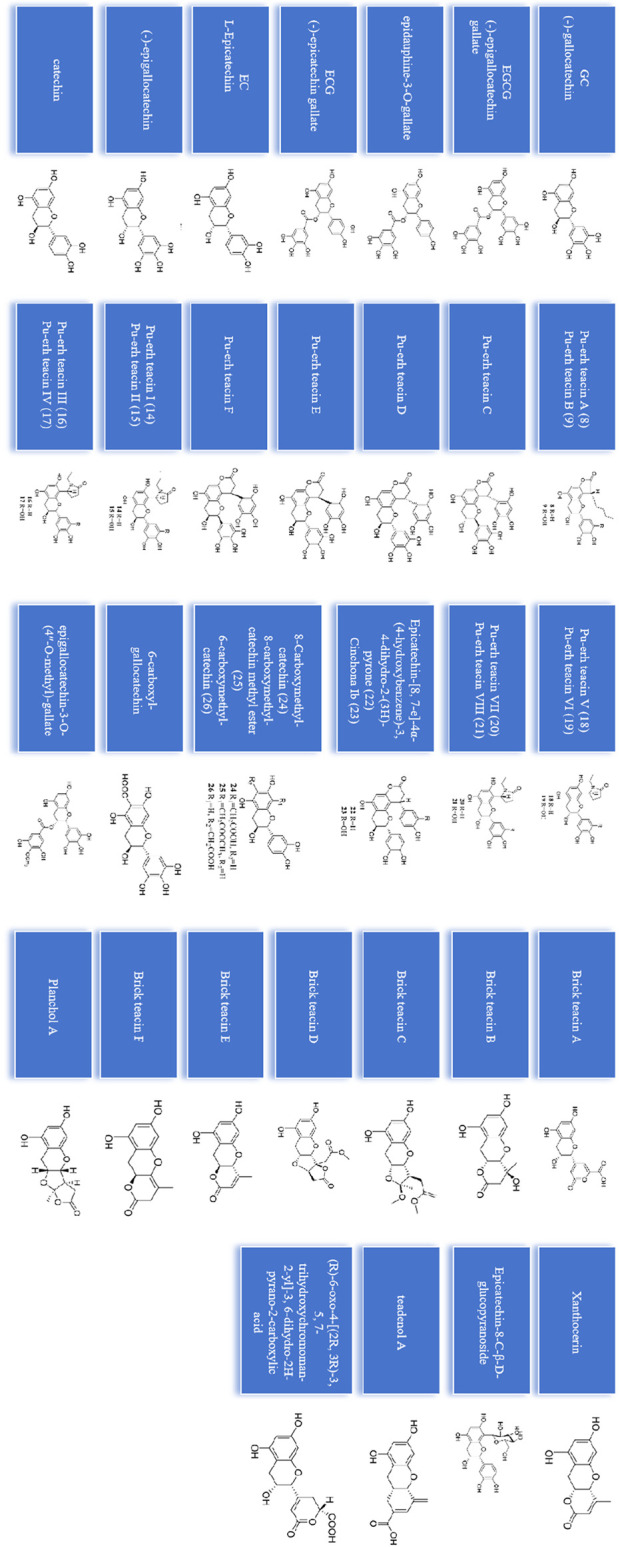
39 catechins identified from oolong tea and black tea.

Catechins are the most important component of tea polyphenols, and different types of fermented tea often contain catechin derivatives with different oxidation configurations. Currently, 39 catechin compounds have been identified from oolong tea and black tea (Hu, 2022) (Figure 2). And a large number of studies have shown that (−)-epigallocatechin-3-gallate (EGCG).

(−)-Epicatechin gallate (ECG) and epigallocatechin (EGC) in fermented tea are the main substances that regulate bone mass and prevent bone loss (Table 3). Nakagawa was the first to study the effect of EGCG on osteoclasts (OCs) and found that after 24 h of intervention, EGCG exhibited a dose-dependent manner (25–100 μM) induced apoptosis of osteoclast like multinucleated cells, while osteoblasts (OBs) were not affected (Nakagawa et al., 2002). Kamon found that 10 mM EGCG and 10 mM ECG can not promoting osteoblast differentiation, but also did not affect the mineralization, meanwhile those extracts reduced the formation of osteoclasts in co culture of differentiated MC3T3-E1 cells and osteoblast (Kamon et al., 2009). Ko treated cells with decaffeinated green tea extract (GTE) and six types of tea polyphenols under osteogenic induction and found that EGC was the most effective in promoting osteogenic differentiation. At 20 μM compared with the control group, EGC significantly increased Alkaline Phosphatase (ALP) levels and Ca^2+^ deposition by 2.3 and 1.7 times, respectively (Ko et al., 2011). This is also the first report on the dual effects of tea polyphenol ECG in promoting osteogenesis and inhibiting the formation of adipocytes in mesenchymal stem cells. Oka found that EGCG inhibits the formation and differentiation of osteoclasts by inhibiting matrix metalloproteinases (MMPs) (Oka et al., 2012). Byun found that ECG stimulates osteoblasts differentiation through Runt related transcription factor 2 (Runx2) and transcriptional co activators with PDZ binding TAZ mediated transcriptional activation (Byun et al., 2014). Deng extracted EGCG from oolong tea and applied tea polyphenols EGCG with concentrations of 10^–5^ and 10^–6^ M to act on human bone marrow stromal stem cells. They found that the intervention group significantly increased the expression level of bone morphogenetic protein-2 (BMP-2) gene (Deng, 2014). They continued to study the role of EGCG as an enhancer or inducer of bone formation the following year, and found that except for BMP2, the activity of ALP and the expression of related osteogenic genes were not affected by EGCG alone treatment (Jin et al., 2015). The research by Xi indicated that EGCG promote osteogenesis in osteoporosis, which is related to the activation of cell cycle protein D1 and the Wnt/β-Catenin signaling pathway (Peng, 2017; Xi et al., 2018). The study by Liu and Lin showed that EGCG pretreatment significantly increased the viability and super oxide dismutase (SOD) activity of osteoblasts through antioxidant effects, while also increasing the expression of osteogenic related genes (Lin et al., 2018; Liu et al., 2018). Kawabata confirmed that EGCG can inhibit insulin like growth factor (IGF-1) induced osteoblast migration through p44/p42 MAP kinase (Kawabata et al., 2018). Chen and Xu found that EGCG reduced the ratio of RANKL/OPG to mRNA expression, ultimately reducing osteoclast activity through the RANK/RANKL/OPG pathway (Chen et al., 2019; Xu et al., 2019). Nishioku found that EGCG inhibits the expression of activated T cell cytoplasmic nuclear factor-1 (NFATc1), which is the main regulatory factor for osteoclastogenesis (Nishioku et al., 2020). Lin discovered that local application of EGCG can enhance the healing process of tibial fractures (Lin et al., 2020). Xu found that EGCG can directly bind to RANK and RANKL and interfere with their interactions, thereby inhibiting RANKL induced IKKalpha/β, Ikappa α, phosphorylation of p65, JNK, ERK1/2, and p38, as well as key downstream regulatory factors, including activated NFATc1, c-Fos, tartaric acid phosphatase (TRAP), c-Src, and tissue protease K in osteoclast (Xu et al., 2021). In addition, Lim indicated that oolong tea extract can exhibit anti-bone loss activity by inhibiting RANKL mediated p38 activation, proving the effectiveness of oolong tea extract in preventing and treating diseases such as osteoporosis. The main component of oolong tea is the polyphenolic compound oolong biflavane B (OFB) (Lim et al., 2020). Wang successfully isolated and cultured hADSCs from adipose tissue, while 5μ mol/L EGCG can enhance its osteogenic differentiation ability (Wang et al., 2021). These studies provide theoretical support for the treatment of osteoporosis with polyphenolic monomers and their derivatives in fermented tea, and some studies also delve into their mechanisms of action (Table 4).

**TABLE 4:** Summary of studies on the inhibition of bone loss by catechins.

References	Extracts	Dosage and timing of use	Affected objects	Outcomes	Country (region)
Nakagawa et al., 2002	EGCG	(25–100 μM) of EGCG for 24 h	OCs, OBs	Induce apoptosis of osteoclast like multinucleated cells, while OBs were not affected	Okohama, Japan
Kamon et al., 2009	EGCG, ECG	10 mM for 10 days	OCs, OBs	Reduced the formation of OCs in co culture of OBs	Ibaraki, Japan
Ko et al., 2011	ECG	20 muM for 7 days and 14 days	BMSCs,OCs	Increased Alkaline Phosphatase levels and Ca^2+^ deposition	Hong Kong, China
Oka et al., 2011	EGCG	10–100 μM for 48 h	OCs	Inhibit the formation and differentiation of osteoclasts by inhibiting matrix metalloproteinases	Showa, Japan
Byun et al., 2014	ECG	10 μM for 6 days	C3H10T1/2 cells	Stimulates OBs differentiation through Runx2	Seoul, Korea
Deng et al., 2014	EGCG	10^–5^ M and10^−6^ M for 21 days	BMSCs	Increased the expression level of BMP-2	Macheng, China
Jin et al., 2015	EGCG	2.5–10 μM for 3, 7, 14 and 21 days	MSCs	The activity of ALP and the expression of related osteogenic genes were not affected by EGCG	Nanning, China
Peng et al., 2017	EGCG	1–50 μM 7、14 for days	OBs	Promote osteogenesis in osteoporosis through Wnt/β-Catenin signaling pathway	Guangdong, China
Xi et al., 2018	EGCG	0.5 mg/kg/day for 4 weeks	OVX mice	Promote osteogenesis in osteoporosis	Beijing, China
Liu et al., 2018	EGCG	5 µM EGCG for 2 h	OBs	Increased the viability and SOD activity of OBs	Shenyang, China
Lin et al., 2018	EGCG	1–10 μM for 24–48 h	BMSCs	Increased the viability and SOD activity of BMSCs	Taiwan
Kawabata et al., 2018	EGCG	0.1–1.0 µM for 24 h	OBs	Inhibit IGF-1 induced osteoblast	Aichi, Japan
Chen et al., 2019	EGCG	20–100 μmol/L for 24 and 48 h	Mouse primary BMSCs	Reduce osteoclast activity through the RANK/RANKL/OPG pathway	Taiwan
Xu et al., 2019	Oxidation derivative of EGCG	10 μM for 4 days	OCs	Reduce osteoclast activity through the RANK/RANKL/OPG pathway	Kunming, China
Nishioku et al., 2020	EGCG	1, 3, 10, and 30 μM for 3 days	OCs, BMSCs	Inhibit the expression of NFATc1	Nagasaki, Japan
Lin et al., 2020	EGCG	10 μmol/L, 40 mL for 2 weeks	Fractured rats	Promote the healing of tibial fractures	Taiwan
Xu et al., 2021	EGCG	10 or 20 μM for 5 days	OCs	Activated NFATc1, c-Fos, TRAP, c-Src, and tissue protease K in osteoclast	Kunming, China
Lim et al., 2020	oolonghomobisflavan B	(1, 2, or 5 μM) of OFB for 3 days	OCs	Exhibit anti bone loss activity by inhibiting RANKL mediated p38 activation	Daegu, Korea
Wang et al., 2021	EGCG	5 μmol/L for 14 days	hADSCs	Enhance osteogenic differentiation ability of EGCG	Guangdong, China

EGCG, (−)-epigallocatechin-3-gallate, ECG, (−)-Epicatechin gallate, BMSCs, Bone marrow mesenchymal stem cells; MSCs, mesenchymal stem cells; OCs, Osteoclasts; OBs, Osteoblasts; OFB, oolonghomobisflavan B, hADSCs, human adipose-derived stem cells, Runx2, Runt related transcription factor 2, BMP-2, bone morphogenetic protein-2; SOD, super oxide dismutase; IGF-1, insulin like growth factor, NFATc1, activated T cell cytoplasmic nuclear factor-1, TRAP, tartaric acid phosphatase; RANKL, receptor activator of nuclear factor kappa-B ligand.

### Tea pigment participate in regulating bone mass

After the fermentation of fermented tea, the tea polyphenols in the tea are significantly reduced, leading to the production of A-ring changing catechins (Pu’er catechins) and a large amount of theaflavins. The tea pigments in black tea mainly include theaflavins (TFs), thearubigins (TRs), theabrownins (TBs). Among them, TFs endow black tea with a relatively bright soup color, making the soup color of black tea appear as a “golden circle”. Due to the positive impact of tea pigment on the quality of black tea, current research mainly focuses on tea pigment. Up to now, it has been proven through testing and analysis that TFs are mainly composed of several types of pigments such as theaflavin-3 (3′)-monogallate, and theaflavin-3-3-digallate in a certain proportion. Many pigments has been found to have a positive effect on bone mass regulation through research (Table 5). Oka was the first to discover that theaflavin-3,3′- migrate (TFDG) can inhibit the formation and differentiation of osteoclasts by inhibiting MMPs, and TFDG may be more effective in inhibiting the formation of actin loops than EGCG (Oka et al., 2012). Liang validated the anti bone loss effects of black tea water extract and thearubigins from both *in vivo* and *in vitro* experiments. The study confirmed that thearubigins, TRs may be one of the functional components of BTE in improving ovariectomy induced osteoporosis in rats and inhibiting *in vitro* osteoclast formation (Liang et al., 2018). Liu showed that TBs significantly inhibited RANKL induced osteoclastogenesis and the expression of related marker proteins, including NFATc1, TRAP, c-Fos, and tissue protease K. *In vivo* studies have demonstrated that treatment with TBs effectively enhances blood biochemical parameters, organ weight and coefficient, femoral bone density, biomechanical properties, as well as bone microstructure in ovariectomized OVX rats (Liu et al., 2018). Gan found that TFDG inhibits RANKL induced osteoclast formation by inhibiting the activation of the ERK signaling pathway and the expression of downstream factors including MMP9 and NFATc1 (Gan, 2018). Ai found that Theaflavin-3, 3′-digallate (TF3) effectively reduces RANKL induced osteoclast formation and reactive oxygen species (ROS) production in a dose-dependent way. Mechanistically speaking, TF3 reduces ROS production and inhibits the mitogen activated protein kinase, MAPK pathway by activating Nrf2 and its downstream heme oxygenase-1 (HO-1) (Ai et al., 2020). Ge found that TFDG significantly increased bone mass in ovariectomized mice. Compared with OVX mice, TFDG reduced the release of pro-inflammatory cytokines and increased the expression of osteogenic markers *in vivo*. *In vitro* experiments have shown that TFDG can promote the formation of osteoblasts in an inflammatory environment and enhance their mineralization ability. During this process, TFDG activates the MAPK, Wnt/beta-Catenin, and BMP/Smad signaling pathways (Ge et al., 2021). Hou found that TF3 can effectively inhibit the expression of pro-inflammatory cytokines in ovariectomized mice, promote polarization of M1 macrophages to M2 macrophages, reduce inflammatory response, promote bone formation, and effectively reduce bone loss. It is expected to become an effective drug for the prevention and treatment of osteoporosis (Hou, 2021).

**TABLE 5:** Summary of research on the use of tea pigments to regulate bone mass.

References	Extracts	Dosage and timing of use	Affected objects	Outcomes	Country (region)
Oka et al., 2012	TFDG	10 and100 μM for 48 h	OCs	Inhibit the formation and differentiation of osteoclasts by inhibiting MMPs	Showa, Japan
Liang et al., 2018	TRs	100 mg/kg body weight for 12 weeks	OVX rats	Improve OVX induced osteoporosis in rats	Kunming, China
Liang et al., 2018	TRs	0–40 ug/mL for 48 h	murine macrophages	Inhibite *in vitro* osteoclast formation	Kunming, China
Liu et al., 2018	TBs	320, 640, 1,280 mg/kg body weight/day for 13 weeks	OVX rats	Improves blood biochemical parameters, organ weight and coefficient, femoral bone density and biomechanical properties	Kunming, China
Liu et al., 2018	TBs	10, 15 or 20 μg/mL for 5 days	OCs	Inhibited RANKL induced osteoclastogenesis and the expression of related marker proteins	Kunming, China
Gan et al., 2018	TFDG	10 mg/kg/day for 8 weeks	OVX mice	Inhibits RANKL induced osteoclast formation by inhibiting the activation of the ERK signaling pathway	Suzhou, China
Ai et al., 2020	TF3	1 mg/kg, 10 mg/kg for 3 months	OVX mice	Reduces RANKL induced osteoclast formation and reactive oxygen species (ROS) production in a dose-dependent way	Shanghai, China
Ai et al., 2020	TF3	1, and 80 mM for 5 days	BMSCs	Reduces ROS production and inhibits the mitogen activated protein kinase, MAPK pathway	Shanghai, China
Ge et al., 2021	TFDG	0–20 μM for 1, 4, and 7 days	BMSCs	Promote the formation of osteoblasts in an inflammatory environment and enhance their mineralization ability	Suzhou, China
Ge et al., 2021	TFDG	1 mg/kg, 10 mg/kg for 5 weeks	OVX mice	Reduced the release of pro-inflammatory cytokines and increased the expression of osteogenic markers	Suzhou, China
Hou, 2021	TF3	0.01–10 μM	BMMs	Inhibit the expression of pro-inflammatory cytokines, promote polarization of M1 macrophages to M2 macrophages, reduce inflammatory response, promote bone formation, reduce bone loss	Suzhou, China

TFDG, theaflavin-3,3′- migrate, TRs, Thearubigins; TBs, Theabrownins, TF3, Theaflavin-3, 3′-digallate, OCs, Osteoclasts; OVX, ovariectomy; BMSCs, Bone marrow mesenchymal stem cells; BMMs, Bone marrow-derived macrophages; RANKL, receptor activator of nuclear factor kappa-B ligand; ROS, reactive oxygen species.

### Other substances participate in regulating bone mass

Fermented tea, due to its fermentation characteristics, has amino acids and trace elements that are more conducive to human absorption and utilization. The composition of free amino acids in tea is closely associated with its quality. Scientific research indicates that fermented tea exhibits a more diverse range of amino acids when compared to non-fermented tea. According to reports, the contents of potassium, magnesium, and calcium are as high as 1980mg/100g, 319mg/100g, and 303mg/100g, respectively, which are considered as key substances for the growth and development of the femur (Song et al., 2020). In addition to substances that are beneficial for bone mass metabolism, researchers have always believed that excessive caffeine intake may have adverse effects on human bone mass. However, Xu found that treatment with caffeine (9.6, 19.2, and 38.4 mg/kg) did not have adverse effects on organ weight, organ coefficient, femur length, bone mineral density, biomechanical properties, or bone microstructure in OVX rats (Xu et al., 2019). Overall, studies have shown that caffeine does not have destructive effects on the skeletal system of OVX rats. When it comes to the long-term consumption of fermented tea leaves, the intake of caffeine is relatively low. Additionally, there are no epidemiological studies that have reported any detrimental effects on bone mass associated with the regular consumption of fermented tea.

### Fermented tea in rodent models: efficiency, safety, and mechanisms

All the aforementioned ingredients demonstrate the bone-protective effect of fermented tea on rodent osteoporosis models. Based on the summary of Table 2 to Table 5, the main rodent research subjects in studies involving fermented tea are rats and mice, with the castrated rat model being the most significant. A 2.5% aqueous black tea extract (BTE) at a dose of 1 mL/100g/day body weight administered daily for 28 days has shown a significant bone-protective effect on castrated rats (Das et al., 2004; Das et al., 2005; Das et al., 2013). Additionally, when comparing different concentrations of black tea extract, it was found that an acute dose of 250–400 mg/kg/day black tea extract demonstrated bone mass protection in castrated rats for 12–16 weeks (Shalan et al., 2017; Wang et al., 2018). The effect was more pronounced at a dose of 400 mg for 16 weeks, leading to improved bone density and reduction in serum inflammatory factors in rats. The use of total black tea extract alone on rodent osteoporosis models suggests that its effect is long-lasting and dose-dependent. Furthermore, currently published studies have not identified any toxic side effects associated with the concentration of black tea extract used. In terms of effective dosage, TPs have shown various degrees of prevention of osteoporosis in ovariectomized rats after intervention with 0.15%–1.5% TPs for 16 weeks. Among these, the intervention effect of 0.5% tea polyphenols has been the most studied and stable (Shen et al., 2008; Shen et al., 2009; Shao et al., 2011; Shen et al., 2011; Shen et al., 2019; Shen and Yin, 2022). Research reports indicate that the acute oral toxicity LD50 of tea polyphenols in male Sprague-Dawley rats is 3.16 g/kg, while in female Sprague-Dawley rats, it is 2.71 g/kg, with a 95% confidence limit of 2.00–3.69 g/kg. By conversion, the daily dosage of 0.5% tea polyphenols is around 100 mg/kg, and there have been no reports of serious toxic side effects associated with this dosage in the study (Yuan et al., 2015). This suggests that continuous administration at this concentration is safe. Further research has been conducted on the main components of tea polyphenols. Continuous administration of 0.5 mg/kg/day EGCG to OVX rats has also shown stability in protecting bone mass, although concentration gradient tests were not conducted (Xi et al., 2018). Regarding the bone mass protection offered by tea pigments, daily doses of 100 mg/kg of TRs (Liang et al., 2018) and 320–1,280 mg/kg of TBs (Liu et al., 2018) have shown a trend towards improving bone mass in OVX rats. In addition, daily doses of 1–10 mg/kg of TF3 (Ai et al., 2020) have shown a trend towards improving bone mass in OVX C57BL mice, with the effect increasing with higher concentrations. Importantly, these administered concentrations of the aforementioned compounds did not reach toxic levels, and their effects were relatively mild and safe. Regarding the mechanisms underlying the protective effect of these substances on rodent bones, a preliminary summary suggests improvements in the balance between osteogenesis and osteoclast activity, enhanced calcium and phosphorus metabolism, promotion of bone immune regulation, modulation of gut microbiota, reduction of oxidative stress associated with osteoporosis, and improvement in endocrine metabolism. These aspects will be further discussed and summarized later.

## The biological mechanism of fermented tea participating in bone mass regulation

### The impact on bone formation

OBs are cells that differentiate from bone marrow mesenchymal stem cells and have the potential to form bone tissue (Figure 2). Currently, many studies have shown that the substances in fermented tea have varying degrees of impact on the osteogenic function of animals and humans. Ko found that 20 μM EGCG significantly increased ALP levels in animal bone tissue by 2.3 times and increased Ca deposition by 1.7 times, while promoting mRNA expression of bone formation markers runt-related transcription factor 2 (Runx2), ALP, OC, and osteopontin (OPN) (Ko et al., 2011). In addition, EGCG can also reduce the differentiation of bone marrow mesenchymal stem cells into adipocytes and inhibit the adipogenic marker PPAR-γ, C/EBP-βand FABPs. It is known that PP1A is crucial for osteoblast differentiation, as inhibiting PP1A activity has been shown to inhibit ECG mediated osteogenic differentiation. Byun found that ECG can effectively stimulate the differentiation of osteoblasts and enhance the nuclear localization of TAZ through the activation of PP1A. The upregulation of osteoblast marker genes is mediated by the increased expression and interaction of transcription co-activators with PDZ binding motifs, TAZ and RUNX2 (Byun et al., 2014). Jin suggests that EGCG can enhance osteogenesis by upregulating BMP2 expression in the presence of bone inducers (Jin et al., 2015). Lin found that EGCG in tea could upregulate the mRNA expression of bone morphogenetic protein 2 (BMP2) and downstream osteogenic-related genes, such as Runx2, ALP, osteonectin, and osteocalcin in animal bone tissue (Lin et al., 2018). Xi indicated that EGCG treatment significantly reduced serum calcium, urinary calcium, body weight, and body fat in mice with secondary osteoporosis, increases leptin levels, and significantly reduces alkaline phosphatase activity (Xi et al., 2018). EGCG also significantly induces cyclin D1, Wnt, and β-catenin expression and inhibition of PPAR-γ expression. Ge found that TFDG can promote the formation of osteoblasts in an inflammatory environment, enhance their mineralization ability, and activate TNF-α, via MAPK, Wnt/β-Catenin and BMP/Smad signaling pathways to promote the transcription of osteogenic related factors such as Runx2 and Osterix, ultimately promoting the differentiation and maturation of osteoblasts (Ge et al., 2021). Lao found that TPs has a huge ability to promote osteogenesis, while upregulating the Runx2-BMP2 mediated osteogenic pathway and inhibiting PPAR-γ to inhibit hADSC differentiation into adipogenic lineages (Lao et al., 2022). These findings emphasize the potential of TPs to combat obesity related osteoporosis. Kawabata found that EGCG can inhibit IGF-I induced osteoblast migration through p44/p42 MAP kinase (Kawabata et al., 2018). These studies indicate that fermented tea has the characteristic of multi-component and multi-target anti osteoporosis, and its promotion of osteogenesis is reflected in the comprehensive effect of compounds.

### The impact on bone resorption

**FIGURE 3:**
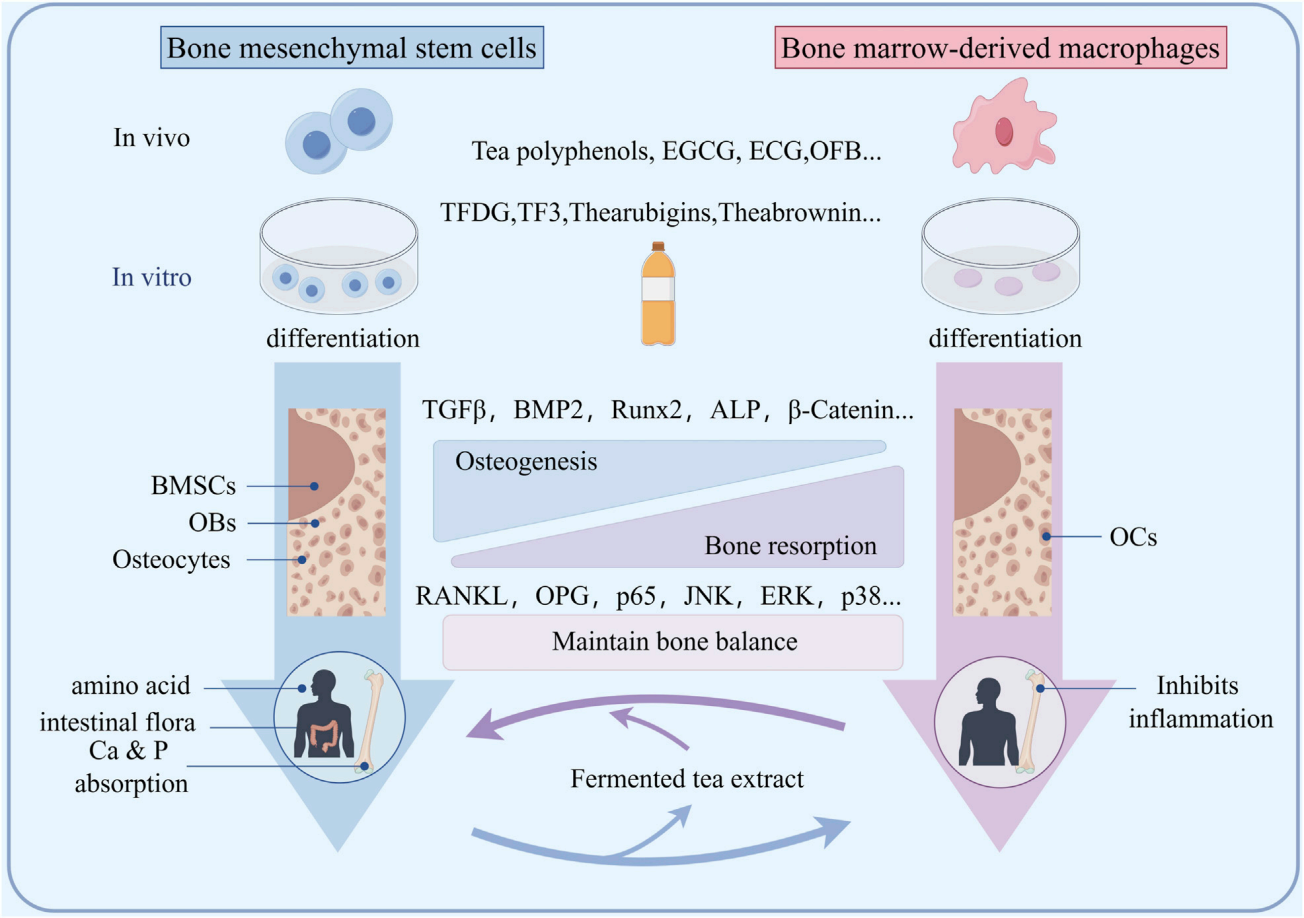
Schematic diagram of the mechanism by which fermented tea extract protects bone loss by maintaining osteogenic osteoclast balance.

Bone resorption mainly refers to the phagocytosis of bone tissue by active OCs. Osteoclasts originate from macrophages in the bone marrow cavity, and their activity is mainly regulated by the RANKL/RANK/OPG pathway (Figure 3). Nakagawa was the first to discover EGCG, which was dose-dependent (25–100 μM) after 24 h of treatment, induce apoptosis and cell death of osteoclast like multinucleated cells, while osteoblasts are not affected (Nakagawa et al., 2002). The Fenton reaction is mainly involved in EGCG induced osteoclast death. Kamon found that ferroptosis is a significant regulatory mechanism used by osteoclasts. Additionally, the Fenton reaction has the potential to effectively manage osteoporosis by modulating ferroptosis in osteoclasts (Kamon et al., 2009). Oka found that the activity of MMP-2 and MMP-9 in rat osteoclast precursor cells treated with TFDG and EGCG was lower than that in the control group. Compared with the control osteoclasts, the MMP-9 mRNA levels in osteoclasts treated with TFDG were significantly reduced. TFDG and EGCG inhibit the formation and differentiation of osteoclasts by inhibiting MMPs, and TFDG may be more effective in inhibiting the formation of actin loops than EGCG (Oka et al., 2012). Xu suggests that TPs can inhibit RANKL induced osteoclastogenesis in RAW264.7 cells and improves ovariectomy induced osteoporosis in rats (Xu et al., 2018). Chen also found that EGCG reduced the RANKL/OPG ratio of mRNA expression and secreted protein levels, and ultimately reduced osteoclast generation through TRAP^+^ staining, and reduced TRAP activity in osteoclasts through the RANK/RANKL/OPG pathway. Moreover, effective concentration can be easily achieved in daily tea drinking (Chen et al., 2019). Wang used an extract of Dendrobium officinale and black tea, which can improve ovariectomy induced osteoporosis in rats and inhibit RANKL stimulated osteoclastogenesis in RAW 264.7 cells (Wang et al., 2018). Xu found that the oxidation product of EGCG, compound 2, binds to RANK with high affinity (KD = 189 nM) and blocks the RANKL-RANK interaction, thereby inhibiting the early RANK signaling pathway induced by RANKL, including p65, JNK, ERK, and p38 in osteoclast precursors (Xu et al., 2019). Nishioku suggested that EGCG can inhibit osteoclast differentiation by downregulating NFATc1 and inhibiting the HO-1-HMGB1-RAGE pathway (Nishioku et al., 2020).

### The impact on calcium and phosphorus metabolism

The normal metabolism of calcium and phosphorus plays an important role in maintaining bone mass balance. Liu suggested that pu’er tea extract (PTE) treatment can maintain calcium and phosphorus homeostasis to varying degrees and improve blood biochemical indicators (Liu et al., 2017). In addition, PTE treatment improved the organ coefficients of the femur, uterus, and vagina, as well as bone density and biomechanical properties of the femur. Xi also indicated that EGCG treatment significantly reduces serum calcium, urinary calcium, body weight, and body fat in mice with secondary osteoporosis, and increases leptin levels (Xi et al., 2018). Das suggests that the protective effect of black tea extract on bone loss is related to the increased activity of two related intestinal mucosal enzymes, namely, alkaline phosphatase (duodenum, jejunum, and ileum) and Ca^2+^ activated ATPase (duodenum, jejunum, and ileum) (Das et al., 2013) (Figure 5). This BTE mediated calcium absorption promotion is combined with an increase in serum estrogen titer and the recovery of all urine, bone, and serum osteoporosis biomarker parameters, including bone histological features (Das et al., 2013).

### Effects on inflammation and immunity

**FIGURE 4:**
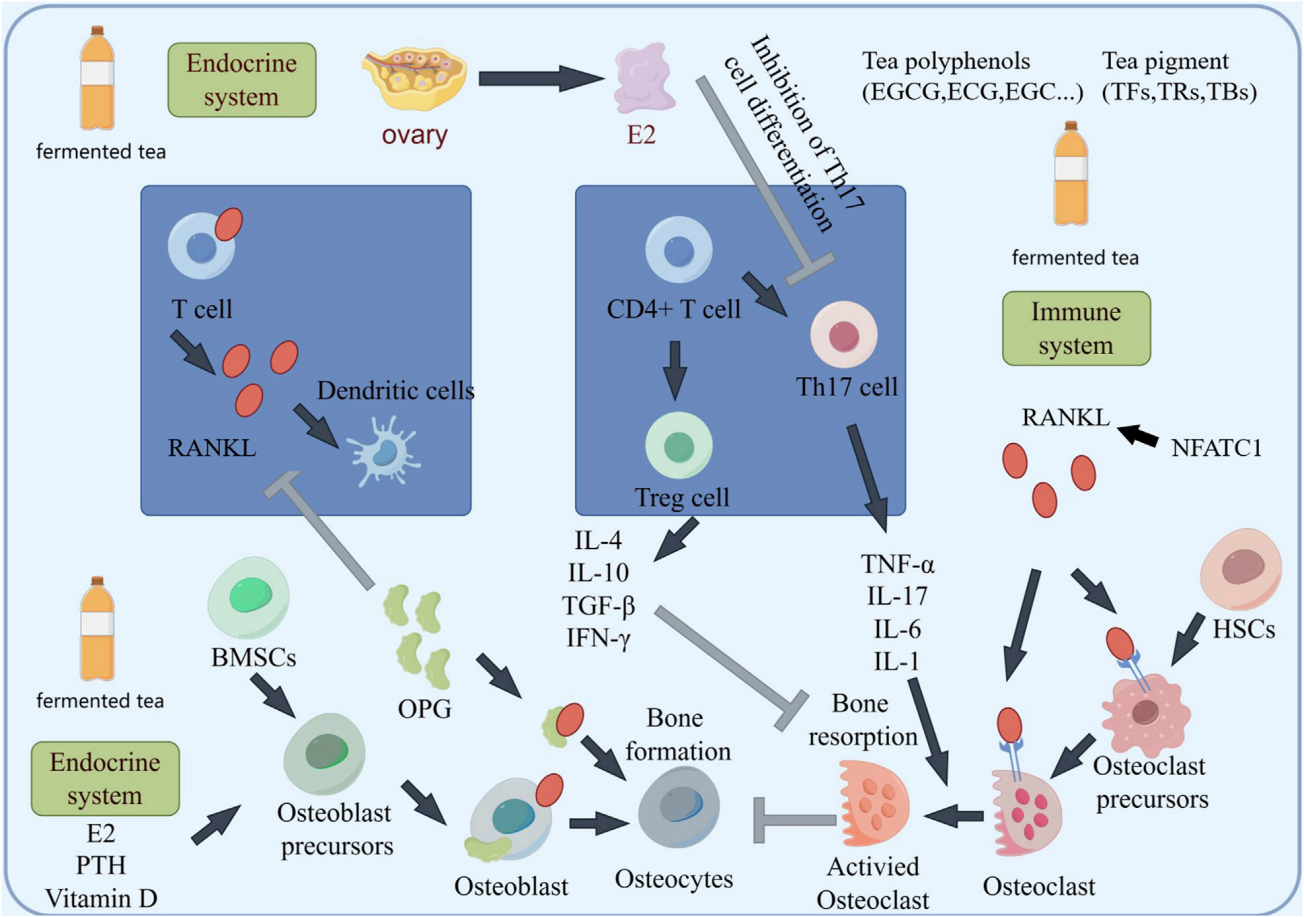
The schematic diagram of fermented tea participating in bone mass regulation through immune regulation suggests that the components in tea may protect bone mass by weakening the activation of Th17 and enhancing Treg differentiation.

**FIGURE 5:**
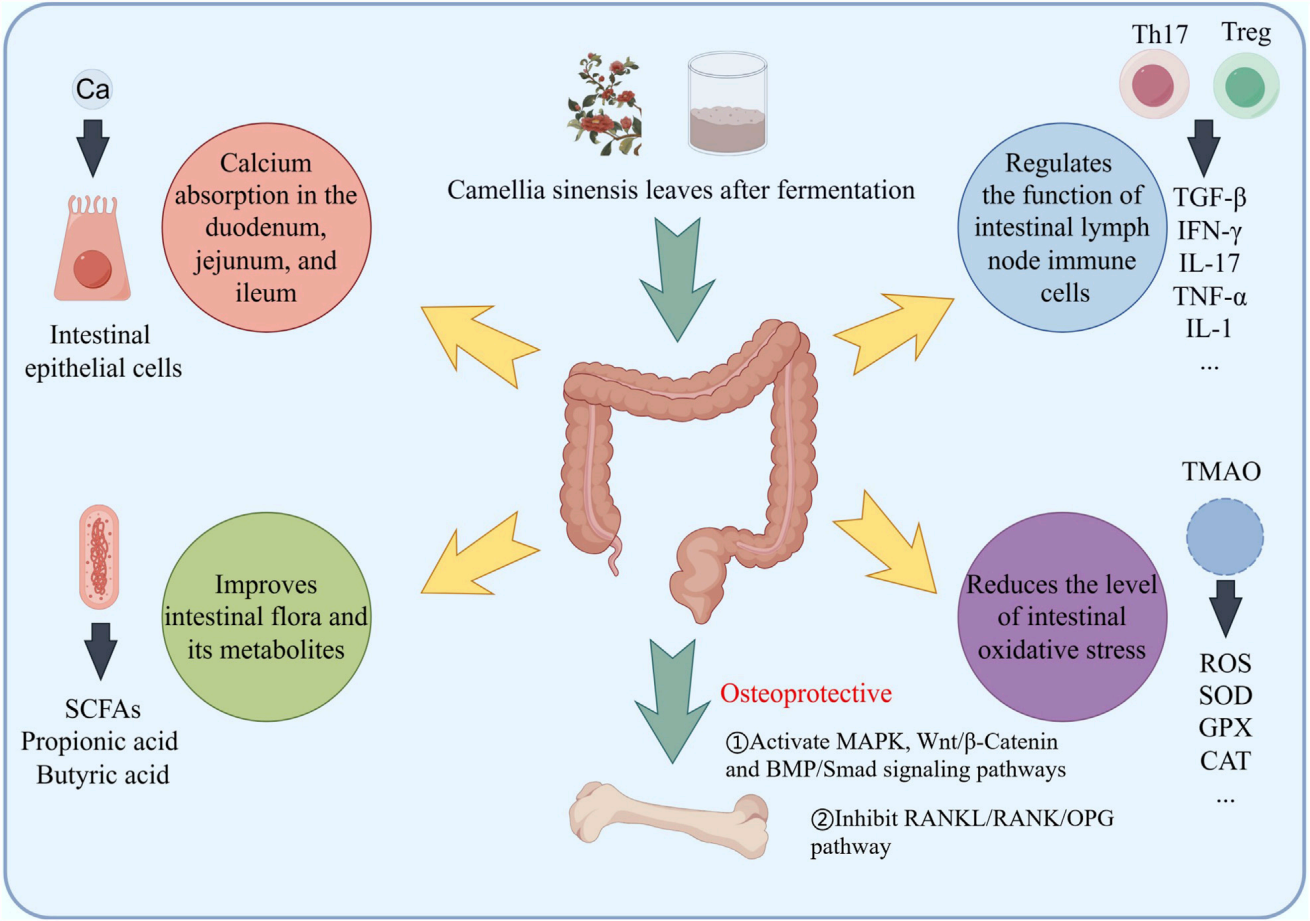
Fermented tea and its active ingredients regulate bone mass through the intestinal pathway, including promoting calcium absorption, regulating gut microbiota, regulating inflammation and immunity, and regulating the production of oxidative stress inducers.

The key factor in the occurrence of osteoporosis is the production of inflammation, in which RANKL activates osteoclast activity and plays an important role (Figure 4). Many immune cells and inflammatory factors can also regulate the activity of osteoclasts, or indirectly affect the content of RANKL in bone tissue. Some inflammatory factors, such as tumor necrosis factor-α (TNF-α), interleukins and others stimulate osteoclast formation and bone resorption by inducing the expression of M-CSF (macrophage colonystimulating factor) and RANKL. Hou found that black tea extract TF3 can reverse some macrophages from M1 phenotype to M2 phenotype and inhibit inflammatory response. TF3 can inhibit pro-inflammatory cytokines TNF-α and IL-1β in osteoporotic mice. And the expression of IL-6 promotes the expression of anti-inflammatory cytokine IL-10 in osteoporotic mice, and this inhibitory/promoting effect is dose-dependent (Hou, 2021). Wang suggested that black tea extract reduces IL-1β, IL-6 levels in OVX rats, improved the organ coefficients of the uterus and femur, as well as BMD (Wang et al., 2018). Due to the activation of NFATc1/c-Fos, it can promote RANKL activation of osteoclasts, while black tea intervention can inhibit NFATc1/c-Fos (Sun et al., 2019; Nishioku et al., 2020). One possible mechanism may be the inhibition of osteoclast proliferation and regulation of NFATC1/c-Fos expression (Figure 4). T Regulatory cell (Treg) and T helper 17 cell (Th17) is an important regulatory cell that regulates inflammation. In recent years, it has been found that the cytokines secreted by Treg/Th17 cells play an important role in bone mass metabolism (Figures 4, 5). Th17 cells secrete TNF-a and IL-17A, promoting inflammation and inducing bone resorption, while Treg secretes TGF-β and IFN-γ, plays a negative immune regulatory role to inhibit bone resorption. Zhang indicated that after treating psoriasis like dermatitis with EGCG, the levels of IL-17A, IL-17F, IL-22, IL-23, and malondialdehyde (MDA) in the plasma of patients decrease. The percentage of CD4^+^ T cells in the composition of spleen immune cells increases. And found an increase in the biological activity of superoxide dismutase, SOD and catalase (CAT) in plasma (Zhang et al., 2016). Yang directly demonstrated that EGCG may induce airway inflammation by increasing the production of IL-10, the number of Treg cells, and increase the expression of Foxp3 mRNA in lung tissue, regulating the proportion of Treg/Th17 at the site of inflammation (Yang and Shang, 2019). However, the impact on Treg/Th17 still needs to be validated in bone tissue.

### Impact on gut microbiota

The gut microbiota affects the occurrence and development of osteoporosis, and its metabolites can serve as biomarkers for the diagnosis and prognosis of osteoporosis (Figure 5). A systematic review of studies on changes in gut microbiota in osteoporosis patients from multiple countries and regions worldwide found that compared to healthy populations, specific bacterial branches such as *Lactobacillus*, Ruminococcus, and Bacteroidetes in the gut of osteoporosis patients were relatively abundant (Blanco, 2020). Studies have shown that there is a significant positive correlation between improved bone condition and increased abundance of short chain fatty acids (SCFAs) producing bacteria. Serum bile acid levels are positively correlated with bone mineral density and biomarkers reflecting bone resorption in postmenopausal women. The regulation of gut microbiota by active ingredients in tea, as well as the decomposition residues of tea components produced by gut microbiota, enable tea to exert anti osteoporosis effects. Research has found that various active ingredients in tea can improve the F/B imbalance in the intestines of high-fat die-induced obese mice, increase the abundance of beneficial bacteria to varying degrees, and change the abundance of *Clostridium*. This genus of bacteria can decompose carbohydrates to produce various organic acids including acetic acid, propionic acid, butyric acid, etc., and participate in lipid and bone mass regulation through SCFAs metabolism (Zhu et al., 2023). It was found that tea polysaccharide regulated intestinal microbiota related metabolites, regulated SCFA-GPCR signal pathway, improved intestinal barrier function and alleviated intestinal microbiota imbalance in high-fat diet-induced obese mice during obesity. SCFAs can inhibit the accumulation of fat in the body, activate the expression of adipocyte cytokines, promote fat consumption, inhibit the expression of lipoprotein lipase (LPL), regulate the triglyceride cycle, and avoid fat accumulation in the blood (Dalile et al., 2019; Zhu et al., 2023). In terms of immune signal regulation, propionic acid and butyric acid inhibit inflammatory factors, including interleukin-6, interleukin-8, and TNF-α. And tea is used to alleviate low-grade inflammation and regulate bone mass.

### The impact on endocrine regulation

Endocrine regulation plays a crucial role in bone mass metabolism, with estrogen being an important factor in promoting osteoblast differentiation and maturation, as well as inhibiting osteoclast activity. Many studies have shown that the components in fermented tea have estrogenic effects in the human body. Das suggested that an increase in serum estradiol levels after supplementing with BTE could significantly reduce bone decay caused by Oophorectomy (Das et al., 2005). Wang indicated that black tea extract can promote the proliferation of ER^+^ cells, and the proliferative effect produced can be antagonized by the estrogen receptor antagonist (Wang, 2018). However, black tea extract showed no significant proliferative effect on ER^−^ cells and ER knockout cells. This indicated that black tea extract has estrogenic effects and is dependent on estrogen receptors. Black tea extract can also increase the phosphorylation level of PI3K, AKT, and ERK, which are used to activate the estrogen signaling pathway. At the same time, it can upregulate the mRNA and protein levels of ER and PGR, and upregulate the mRNA levels of downstream target genes PS2 and cyclin D1 of ER. Excessive glucocorticoids can inhibit the activity of bone marrow mesenchymal stem cells, and many patients may experience complications such as osteoporosis and femoral head necrosis after long-term use of glucocorticoid drugs. Zhao found that after intervention with bone marrow mesenchymal stem cells combined with theaflavin in hormone induced femoral head necrosis in rats, a large amount of new bone formation appeared in the damaged femoral head area, and the adipocytes in the femoral bone marrow were regular, indicated that theaflavin can antagonize bone destruction caused by excessive glucocorticoids and promote osteogenic differentiation of bone marrow mesenchymal stem cells (Zhao et al., 2015). Previous research has indicated that estrogen deficiency can lead to aberrant expression of Foxp3 and RORγt, thereby affecting the Th17/Treg equilibrium (D'Amelio et al., 2008; Chen et al., 2015). In the context of estrogen deficiency, Treg cells can synthesize and secrete IL-10, which promotes an increase in OPG content in osteoblasts (Figure 4). This mechanism is also a microcosm of how fermented tea affects osteoclast activity by influencing estrogen and then regulating Th17/Treg balance.

### The impact on oxidative stress

Oxidative stress can disrupt the balance between antioxidant capacity and ROS production. ROS mainly comes from the production of mitochondria and NADPH oxidase (NOXs). Among them, NOX2 and NOX4 play important roles in bone metabolism. Macrophage colony-stimulating factor (M-CSF) instantaneously increases intracellular ROS levels through NOX2 and induces activation of extracellular signal regulated kinases and expression of RANK in OC precursor cells, increasing bone resorption. NOX4 is upregulated during the differentiation of BMSCs into osteoblasts, reducing bone formation and promoting the development of OP. Nrf2 is a transcription factor involved in oxidative stress. In rat osteoblasts, upregulation of Nrf2 promotes the activity of antioxidant enzymes such as SOD, glutathione peroxidase (GPX), catalase (CAT), etc., weakens oxidative stress, promotes OBs differentiation and bone formation (Figure 4). Vester et al. found that low-dose TE stimulation of primary human osteoblasts can improve mineralization during 21 days oxidative stress induced by H_2_O_2_. In addition, during the differentiation process of osteoblasts, BTE supplementation combined with H_2_O_2_ leads to the increasing expression of osteocalcin and collagen 1α (Vester et al., 2014). Ai found that black tea extract TF3 reduces ROS production and inhibits the mitogen activated protein kinase, MAPK pathway by activating Nrf2 and its downstream heme oxygenase-1 (HO-1) (Ai et al., 2020). Trimethylamine N-oxide (TMAO) is one of the common metabolites produced by the gut microbiota and has become a risk factor for osteoporosis. Elevated serum levels of trimethylamine oxide in postmenopausal women can increase their risk of osteoporosis and fractures. TMAO can activate ROS dependent NF-κB signaling pathway, and promotes the differentiation of OCs, leading to bone loss. Chen found that oolong tea extract reduced the mRNA levels of vascular inflammation markers, such as TNF-α, Vascular cell adhesion molecule-1 (VCAM-1) and E-selectin in mouse (Chen et al., 2019). Analysis of gut microbiota and metabolites suggested that the oolong tea extract treatment group upregulated the genus *Lactobacillus*, and the compounds in it, significantly reduced plasma TMAO (Figure 5).

## The industrial application potential of fermented tea

### Extraction and application of polyphenolic

**FIGURE 6:**
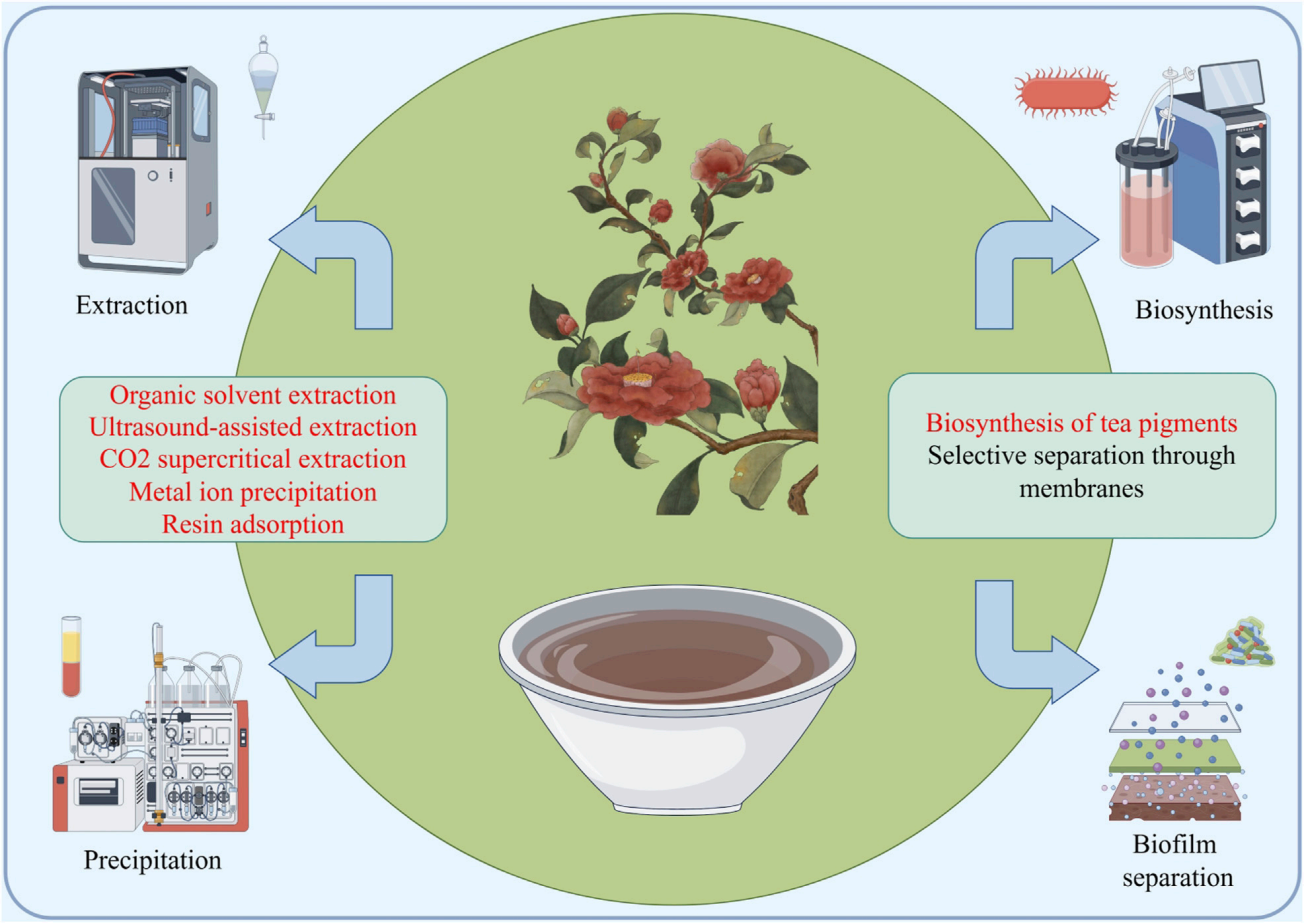
A schematic diagram of the industrial application potential of fermented tea, with the main synthesis and extraction methods focused on traditional methods and supercritical extraction, membrane separation, precipitation, biosynthesis, etc.

At present, tea polyphenols have attracted the attention of many scientists both domestically and internationally. Through continuous exploration by numerous domestic and foreign scholars, the extraction and separation technology of tea polyphenols has been effectively improved (Figure 6). The most commonly used method is solvent extraction, which can be further divided into water extraction method and organic solvent extraction method according to the different extraction liquids. The extraction rate of water extraction method is generally around 5%. Compared with other extraction methods, the product purity of aqueous extraction method is low and contains a large amount of impurities, and the oxidation degree of extracted tea polyphenols is also high. The extraction rate of organic solvent extraction method is about 7%. Compared with aqueous extraction method, the oxidation degree of tea polyphenols extracted with organic solvent is relatively low, but it also has the disadvantage of high impurity content (Beata et al., 2007). Compared with other methods for extracting tea polyphenols, solvent extraction has the advantages of low cost, simple operation, and easy control of production conditions. However, the experimental process takes a long time and it is difficult to separate and purify tea polyphenols in the later stage. Multiple distillation and multi-step processes are required to obtain relatively high purity tea polyphenols. In addition, excessive solvent consumption during the preparation process makes it difficult to recover and reuse, which can easily cause environmental pollution.

The metal ion precipitation method utilizes the phenomenon of tea polyphenols easily forming complex deposits with certain metal ions under specific conditions, and obtains the final product tea polyphenols through processes such as centrifugation, acid dissolution, and extraction (Yu and Wang, 2001; Hu, 2011). At present, there are mainly three types of precipitants: heavy metal alkaline salt precipitants, hydroxide precipitants, and metal ion precipitants (Yi, 2013). Although ion precipitation method has the advantages of strong selectivity and high purity compared to other extraction methods, the precipitant used is harmful to human health. Therefore, in practical applications, it is necessary to strictly control the content of organic solvents. If there are metal salts remaining in the product, it will pose a great threat to product safety.

Ultrasonic assisted extraction method refers to the use of the unique mechanical, cavitation, and thermal effects of ultrasound during the extraction process to greatly improve the compression and stretching efficiency of the material when it propagates in the liquid. At the same time, the huge shock wave and shear force generated by the mechanical crushing and cavitation of ultrasound break the material cells, thereby increasing the dissolution rate and quantity of polyphenolic substances, promote the release, diffusion, and dissolution of tea polyphenols within tea cells, thereby obtaining relatively pure tea polyphenols (Li et al., 2017; Bao et al., 2018). The ultrasonic assisted extraction method has a simpler process compared to other extraction methods, lower oxidation loss of tea polyphenols, and higher product yield. However, in the process of using ultrasonic assisted extraction to extract tea polyphenols, the ultrasonic wave will be weakened, resulting in a change in the effective area of the ultrasonic wave and the formation of an ultrasonic blank area, which will affect the extraction efficiency of tea polyphenols. At the same time, the cost of ultrasonic assisted extraction is also very expensive, which limits the use of this method.

Supercritical extraction, as a new type of chemical separation technology, has been widely applied in various fields for a long time (Zhang et al., 2019). Supercritical fluid extraction technology refers to the process of effectively separating, extracting, and purifying the matrix and extract through the influence of pressure and water temperature on the solubility of supercritical fluids (Wang et al., 2019). In the process of extracting substances, supercritical CO_2_ is generally used as the main extraction solvent. Due to the strong solubility and high extraction ability of supercritical CO_2_, this method plays an irreplaceable role in various fields. Li used ssupercritical CO_2_ (SCF-CO_2_) for the extraction of tea polyphenols from tea leaves. The extraction process involved setting the CO_2_ pressure at 25 MPa and the extraction temperature at 80°C. Supercritical fluid extraction was performed on the tea leaves for a duration of 2.5 h (Li, 2009).

Membrane separation method refers to the separation of various substance components in a solvent by selecting a suitable selective permeation membrane as the isolation medium and utilizing different potential differences, concentration differences, and pressure differences at both ends of the selective membrane. When this technology is applied in the extraction process of tea polyphenols, it first uses ultrafiltration and microfiltration membranes to remove impurities and clarify substances, and then uses reverse osmosis and nanofiltration membranes to concentrate and obtain tea polyphenol products. Research has confirmed that the retention rate of tea polyphenols by reverse osmosis membranes is about 10% higher than that of nanofiltration membranes. In practical applications, membrane separation method has lower extraction efficiency than other methods, and there are still certain difficulties in practical operation (Sun et al., 2009). It is necessary to strictly control the selection of different membrane materials, and the filtration efficiency is low. The refining process is relatively complex, and other separation and purification techniques need to be combined to obtain high-quality tea polyphenols. Therefore, using membrane separation method to extract tea polyphenols is limited and cannot be widely used.

Resin has excellent selective separation properties, which can effectively adsorb tea polyphenols, achieve effective separation of tea polyphenols from other different substances, and thus extract and purify tea polyphenols. Niu selected the most suitable resin for separation and purification from eight different types of resins, and used this resin to adsorb and separate tea polyphenols. The extraction rate of tea polyphenols can reach about 80%, and the purity can reach 98%. Compared with other methods, the resin adsorption separation method for extracting tea polyphenols has a relatively simple process, high extraction efficiency, and does not cause environmental pollution, which is very in line with the concept of green chemistry. However, this method has very strict requirements for resin, and the cost of resin is high (Niu et al., 2017). At the same time, the resin is prone to deactivation during operation, causing blockages and low extraction efficiency. Therefore, the resin adsorption method cannot be used on a large scale.

## Extraction and synthesis process application of tea pigments

### Extraction process of tea pigments

The solvent extraction method is the most commonly used method for extracting tea pigments, which utilizes the good solubility of tea pigments in the extraction solvent to achieve the extraction and separation of tea pigments. At present, the commonly used solvents for extracting tea pigments include ethanol, ethyl acetate, acetone, purified water, and n-butanol. Shang et al. used black tea sold in the China market as the extraction material, and used various extraction solvents such as ethyl acetate, benzene, ethanol, acetone, purified water, methanol, and multiple extraction temperatures and times to extract tea pigments from black tea, in order to explore the optimal extraction scheme. The research results show that under room temperature conditions, using purified water as the extraction solvent and maintaining the extraction time for 10 min, the extraction rate of tea pigments in black tea is the highest, at 21.5% (Shang et al., 2014). Liu used black tea sold in the Yingde market in Guangdong as materials, ethanol as the extraction solvent, and microwave-assisted extraction of tea pigments. Through experimental research, it was found that ethanol concentration, extraction time, solid-liquid ratio, and microwave power are four important factors affecting the extraction rate of black tea pigments. When ethanol concentration is 40%, extraction time is 5 min, solid-liquid ratio (m/v) is 1:25, and microwave power is 490 W, The extraction effect of tea pigments is the best, with an extraction rate of 21.78% (Liu et al., 2013).

### The synthesis process of tea pigments

The *in vitro* simulated oxidation of tea pigments refers to the artificial creation of conditions or environments that can promote the oxidation reaction of tea polyphenols, thereby simulating the process of tea polyphenols in black tea undergoing oxidation reaction and transforming into tea pigments. According to different operating principles, this method can be further divided into three methods: *in vitro* simulated enzymatic oxidation, *in vitro* simulated chemical oxidation, and autooxidation. Xia Tao et al. prepared a suspension fermentation system for black tea using fresh tea leaves as materials, and gave the system a certain temperature, oxygen, and pH. After a certain period of fermentation, it was found that when the temperature of the fermentation system was maintained at 28.5–29.0°C, the oxygen flux was maintained at 13.0–15.0 mL/min, the pH was maintained at 4.6–4.8, and the fermentation time was maintained at 55.5–58.9min, the fermentation effect was optimal. The accumulation of TFs and TRs in the fermentation system was the highest (Xia and Gao, 1999). On the basis of comparing the enzyme activity of fresh tea leaves from 22 tea tree varieties, Yu uses *in vitro* simulated enzymatic oxidation method to ferment different catechin components using high enzyme activity tea leaves and exogenous enzymes as enzyme sources. The experimental results showed that different catechin components showed a gradually decreasing trend after a period of fermentation, the accumulation of TFs in the fermentation system shows a trend of first increasing and then decreasing (Yu, 2016). It is speculated that the reason for the above trend of changes is that catechins in the fermentation system first undergo enzymatic oxidation reaction under the action of enzymes, generating fermentation products such as TFs. Therefore, at the beginning, as the content of catechins decreases, the accumulation of TFs will actually increase. Later, with the extension of fermentation time, the generated TFs will further oxidize to form fermentation products such as TBs, ultimately leading to a gradual decrease in the accumulation of TFs. The effective way to prevent this trend from occurring is to control the fermentation time reasonably.

In addition, the latest research indicates that Zhou immobilized tyrosinase using catechins as crosslinking agents, and applied them to the synthesis of theaflavin-3,3′-diglycerides. Crosslinked enzymes exhibit superior catalytic performance (thermal stability, organic solvent tolerance, substrate tolerance) compared to free enzymes. When cross-linked enzymes are used to synthesize theaflavin-3,3′-diglycerides, the mass concentration of the product can reach 800 μG/mL, and the cross-linked enzyme can be reused for at least three batches (Zhou et al., 2023). Using this method to prepare theaflavins can significantly reduce the application cost of the enzyme and has potential industrial application value.

## Summary

Fermented tea extract and its compound components, there is currently evidence to suggest the benefits of drinking tea for people with osteoporosis. Based on this, many studies have been conducted on the dosage, exact ingredients, mechanisms, and industrial applications of protecting osteoporosis. In terms of dosage, for semi-fermented oolong tea, drinking at least one cup (250 mL) of oolong tea per day has a protective effect on bone mass; Black tea, on the other hand, may achieve bone protective effects in even fewer doses (Chen et al., 2019). The components that play a major role in bone metabolism in fermented tea are investigated. Firstly, the substances represented by tea polyphenols include catechins and flavonoids; Among them, EGCG and ECG are the main active ingredients for anti osteoporosis, with their targets focused on inhibiting bone resorption and promoting bone formation (Wang, 2020); As a unique substance in fermented tea, tea pigments are also considered an important component for stable bone protection in fermented tea. Among them, Theaflavin-3,3′- digalate, represented by theaflavins, is the most studied component, which has been proven to have strong bone mass regulating activity in both *in vivo* and *in vitro* experiments (Hou, 2021). Other substances in fermented tea, such as bisflavane B and tea polysaccharides, have also been proven to have anti osteoporosis activity, while amino acids, trace elements also have the effect of promoting bone formation. This is mainly as a raw material for bone formation. Fermented tea is a good nutritional supplement. In addition, there have been epidemiological reports that caffeine in tea has a negative effect on bone mass protection, but animal experiments have shown that long-term use of caffeine has no significant effect on bone density. However, for people who are sensitive to caffeine, long-term and excessive consumption may cause insomnia and palpitations, which can lead to endocrine disorders. This may be a negative factor for osteoporosis. Therefore, we recommend that such people use low caffeine fermented tea or other bone protection products to achieve the goal of protecting bone mass (Xu et al., 2019).

In terms of mechanism research, we found that fermented tea exerts anti osteoporosis effects through the following pathways: 1) It can regulate the differentiation direction of bone marrow mesenchymal stem cells through MAPK, Wnt/beta Catenin, and BMP/Smad signaling pathways, promote the expression of osteogenic genes such as Runx2 and ALP, inhibit fat differentiation, and promote osteoblast migration to supplement lost bone through IGF-1 mediation (Peng, 2017). 2) It can also inhibit the formation and differentiation of osteoclasts by inhibiting MMPs, downregulating NFATc1, and inhibiting the HO-1-HMGB1-RAGE pathway. In addition, it can reduce the activity of osteoclasts through the RANK/RANKL/OPG pathway (Oka et al., 2012; Chen et al., 2019). 3) Fermented tea can maintain calcium and phosphorus homeostasis to varying degrees, which is related to enhancing the activity of two related intestinal mucosal enzymes, namely, alkaline phosphatase and Ca^2+^ activated ATPase, thereby promoting calcium absorption (Das et al., 2013). 4) Fermented tea can reduce the levels of inflammatory factors, such as TNF-α, IL-17, IL-1, IL-6, etc., while maintaining bone immune balance by regulating Treg/Th17 cell balance (Yang and Shang, 2019). 5) Fermented tea can regulate gut microbiota and its metabolites, and protect against bone loss through the SCFA-GPCR signaling pathway and immune signaling pathway (Zhu et al., 2023). 6) Fermented tea has estrogenic effects that can increase ERα to activate the estrogen signaling pathway and increase the phosphorylation levels of downstream PI3K, AKT, and ERK, and is regulated by the level of key proteins phosphorylation; Simultaneously, it has a repairing effect on bone damage caused by glucocorticoids (Zhao et al., 2015); 7) The extract of fermented tea can promote the activity of antioxidant enzymes such as SOD, GPX, CAT. through the upregulation of Nrf2, weaken TMAO mediated oxidative stress, promote OBs differentiation and bone formation (Chen et al., 2019).

## Prospect

For the application prospects of fermented tea in anti osteoporosis, the extraction and synthesis of active ingredients for anti osteoporosis is a major direction (Niu et al., 2017). The tea polyphenols have been studied by scientists around the world and various methods were found to quickly and effectively extract and separate these components, such as solvent extraction, metal ion precipitation, ultrasonic assisted extraction, supercritical extraction, membrane separation, resin adsorption separation method. The unique component of tea pigments in fermented tea, in addition to traditional extraction methods, is also widely used through biosynthesis methods such as *In Vitro* Simulated Oxidation (Yu, 2016). Futhermore, the development of biological enzyme synthesis has also promoted the progress of the tea pigment synthesis industry, which provides support for the use of fermented tea as an anti osteoporosis agent (Zhou et al., 2023).
